# Case report: Intraretinal hyperflow microinfiltration lesions on swept-source optical coherence tomography angiography as a potential biomarker of primary vitreoretinal lymphoma

**DOI:** 10.3389/fmed.2024.1386979

**Published:** 2024-04-26

**Authors:** Zhangxing Xu, Haixia Bai, Xin Liu, Junhui Shen, Yongchao Su, Yao Wang

**Affiliations:** ^1^Eye Center, The Second Affiliated Hospital, School of Medicine, Zhejiang University, Zhejiang Provincial Key Laboratory of Ophthalmology, Zhejiang Provincial Clinical Research Center for Eye Diseases, Zhejiang Provincial Engineering Institute on Eye Diseases, Hangzhou, Zhejiang, China; ^2^Department of Ophthalmology, Ningbo Medical Center LiHuiLi Hospital, Ningbo, Zhejiang Province, China; ^3^Department of Ophthalmology, Haiyan People's Hospital, Jiaxing, Zhejiang Province, China

**Keywords:** primary vitreoretinal lymphoma (PVRL), swept-source optical coherence tomography angiography (SS-OCTA), vertical hyperreflective lesions (VHRLs), Intraretinal hyperflow microinfiltration lesions, biomarker

## Abstract

Primary vitreoretinal lymphoma (PVRL) is often associated with central nervous system involvement, contributing to a heightened mortality rate, thus imaging features that are characteristic enough to be potential biomarkers of PVRL are important, either in diagnosis or in assessment of disease activity. This report details the case of a 68-year-old male who presented with blurred vision in both eyes persisting for 2 months. Fundus examination demonstrated vitreous opacity and multiple subretinal yellow nodular lesions of varying sizes in the peripheral fundus of both eyes. Multiple vertical hyperreflective lesions in the neural retina of posterior pole, indistinct outer retina borders in the fovea, and hyperreflective lesions in the sub-retinal pigment epithelium (RPE) space of the peripheral retina were demonstrated on swept-source optical coherence tomography (SS-OCT) of the left eye. Hyperflow signals corresponding to the vertical hyperreflective lesions were detected on swept-source optical coherence tomography angiography (SS-OCTA) images of retinal deep capillary plexus (DCP) layer. Notably, the hyperflow signals, precisely located around retinal vessels from the nerve fiber layer to the outer plexiform layer, were postulated to stem from the dilation of infiltrated retinal vessels. Vitreous pathological results of the left eye confirmed the diagnosis of PVRL. Treatments with intravitreal methotrexate injections led to a marked improvement of best-corrected visual acuity (BCVA) and regression of the hyperflow microinfiltration lesions demonstrated on SS-OCTA. In conclusion, SS-OCTA effectively delineated the vertical hyperreflective lesions and corresponding hyperflow signals in the posterior pole macular region of a patient with PVRL. These lesions significantly diminished following intravitreal methotrexate injections. We speculated that the specific hyperflow signals on SS-OCTA could act as a potential biomarker of PVRL, and SS-OCTA holds promise in facilitating early diagnosis and monitoring therapeutic responses in PVRL cases.

## Introduction

Primary vitreoretinal lymphoma (PVRL) is a rare malignancy occurring in the retina or vitreous of the eye, comprising less than 0.01% of all ocular diseases (1–4). The average onset age of PVRL is 50–60 years, with a male-to-female ratio of 1: 2. It is a high-grade typical B-cell malignancy. Central nervous system involvement will appear in 50–80% of patients with PVRL several years after ocular symptoms, which causes a poor prognosis (2, 5). The etiopathogenesis of primary intraocular lymphoma is still not well understood (6). Both the infectious theory and hematological spread theory have been implicated in the etiology of PVRL (7). Patients typically present with nonspecific complaints of painless blurred vision, floaters or both. Examination of the posterior segment reveals vitritis and development of creamy lymphoma choroidal infiltration with orange-yellow infiltrates deep to the RPE (4, 8). These creamy lesions can also develop in the posterior pole of retina. Optical coherence tomography (OCT) offers an obvious advantage in assessing disease activity and monitoring response to treatment in PVRL. OCT demonstrates vitreous cells, RPE nodularity, and outer retinal hyperreflectivity, pigment epithelial detachment, epiretinal membrane and retinal disorganization (9). Vertical hyperreflective lesions are also a common finding on OCT in PVRL (10). As one of the few reports on optical coherence tomography angiography (OCTA) presentations of PVRL, the research of Chen et al. discovered perivascular flower-bud-like lesions (PFBLs) visualized on *enface* OCTA as novel features of PVRL, which may represent the perivascular infiltration or migration of lymphoma cells (11). This report elucidates the characteristics of vertical hyperreflective lesions on swept-source OCT/OCTA (SS-OCT/OCTA), analyzing the association between vertical hyperreflective lesions on SS-OCT and corresponding hyperflow signals on SS-OCTA images of deep capillary plexus (DCP) layer. Therefore, we try to delve into the possible origin of the hyperflow signals, and consolidate the role of SS-OCTA findings in diagnosing PVRL and monitoring the activity of PVRL involving the posterior pole of fundus.

## Case presentation

**Figure 1:**
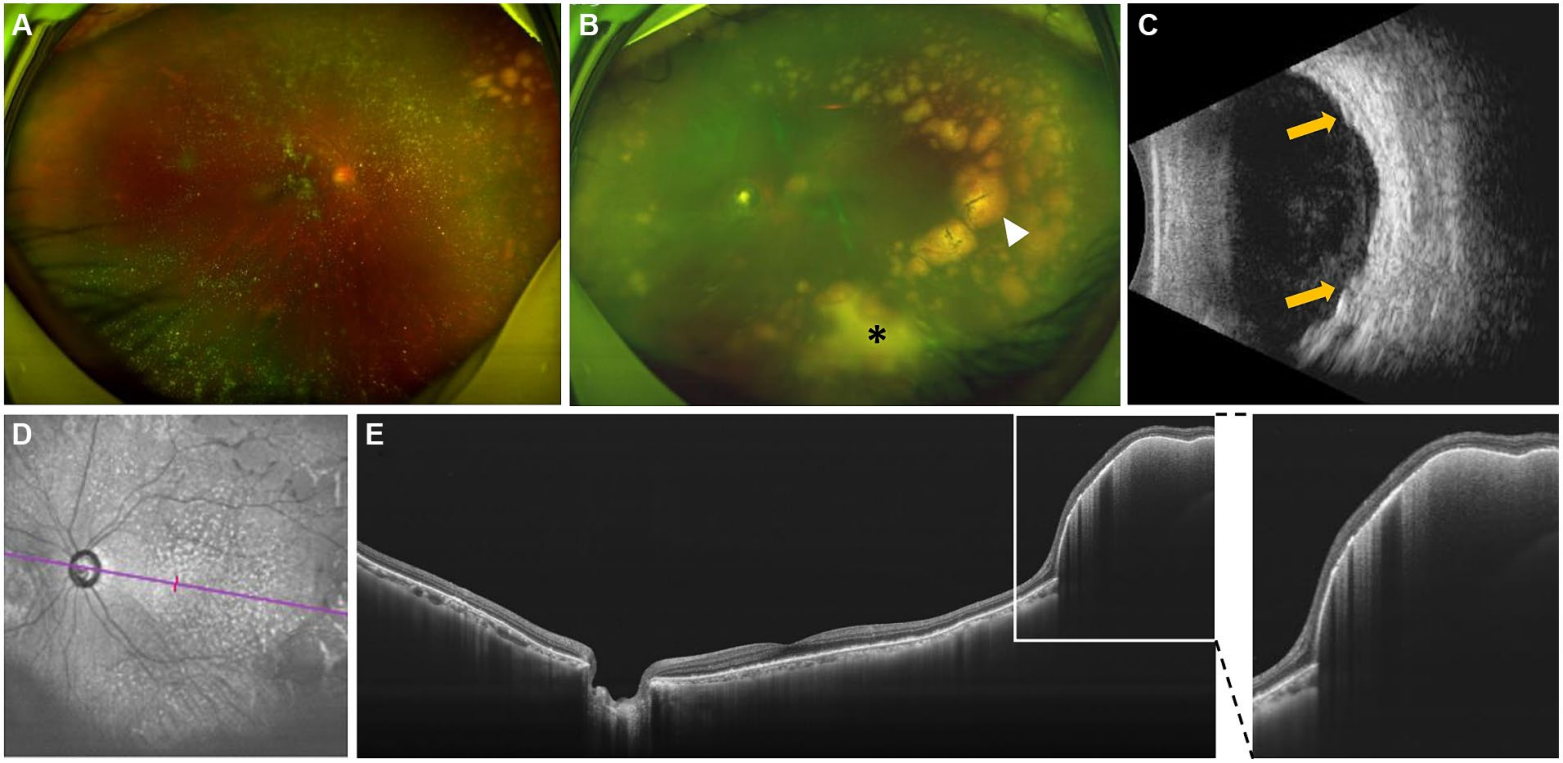
Fundus photographs, B-scan ultrasonography and swept-source Optical Coherence Tomography (SS-OCT) examinations at initial presentation. **(A)** Asteroid hyalosis and several subretinal yellow deposits were detected in the nasal retina of the right eye. **(B)** Moderate vitreous opacity, multiple subretinal yellow nodular lesions with varying sizes, streaky pigmentation (white arrowhead) and white exudation (asterisk) on the surface of several yellow subretinal nodules were detected in the left eye. **(C)** B-scan ultrasonography showed clusters of moderately or highly condensed punctate echoes in vitreous cavity, and irregular hypoechoic lesions (yellow arrows) under the retina of the left eye. **(D,E)** Infrared image and SS-OCT images with scanning length of 24 mm of the left eye. Homogenous hyperreflective lesions in the sub-RPE space were detected in the peripheral retina. An enlarged inset is provided on the right.

**Figure 2:**
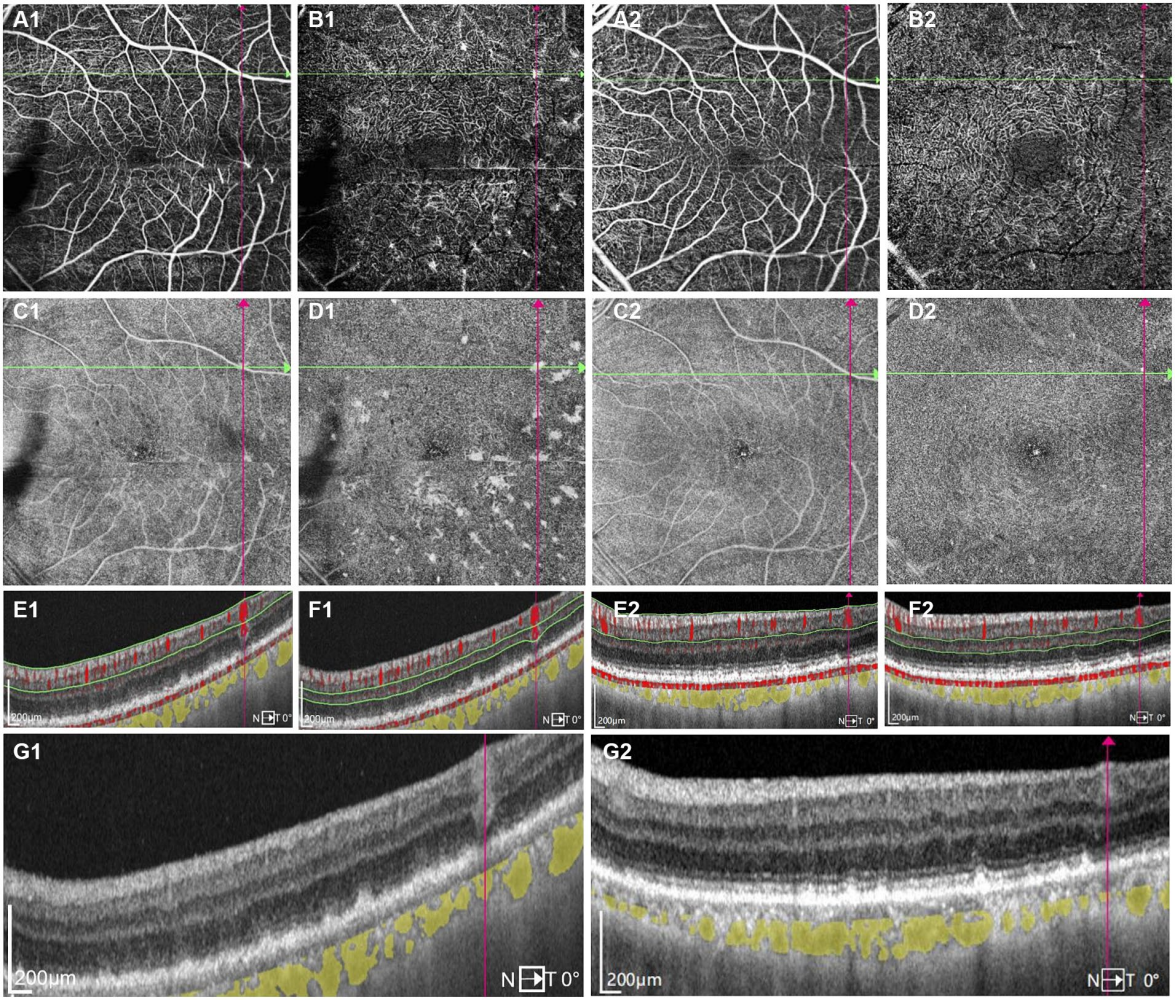
One typical intraretinal hyperflow microinfiltration lesions located beneath superficial retinal vessel on Swept-Source Optical Coherence Tomography Angiography (SS-OCTA) with scanning areas of 6 mm × 6 mm of the left eye **(A1–G1)** before and **(A2–G2)** after induction phase of intravitreal methotrexate injections. **(A1)** SS-OCTA image of (superficial capillary plexus) SCP layer showed no abnormal blood flow signals correlated with lymphoma. **(B1)** SS-OCTA image of retinal deep capillary plexus (DCP) layer showed several hyperflow spots. **(C1)** *Enface* SS-OCTA structural images of SCP layer showed no abnormal reflectance correlated with lymphoma. **(D1)** *Enface* SS-OCTA structural images of DCP layer showed multiple hyperreflective lesions. **(E1,F1)** SS-OCT B-scan with flow overlay of **(E1)** SCP layer and **(F1)** DCP layer showed inhomogenous flow signals of the vertical intraretinal hyperreflective lesion. The boundary of flow signals overlaid on the vertical intraretinal hyperreflective lesions was difficult to clearly distinguish from the flow signals of retinal vessels above them. **(G1)** SS-OCT showed outer retina with fuzzy borders and the vertical hyperreflective lesion extended from retinal nerve fiber layer to RPE. The hyporeflective vascular wall boundary of vessels above intraretinal infiltrations was absent. **(A2)** SS-OCTA image of SCP layer showed no great difference from **A1**. **(B2)** SS-OCTA image of DCP layer showed the preexisting hyperflow spots almost disappeared. **(C2)** *Enface* SS-OCTA structural images of SCP layer showed no great difference from **C1**. **(D2)** *Enface* SS-OCTA structural images of DCP layer showed the preexisting hyperreflective lesions almost disappeared. **(E2,F2)** SS-OCT B-scan with flow overlay of **(E2)** SCP layer and **(F2)** DCP layer showed the preexisting hyperflow signals of the vertical intraretinal hyperreflective lesion almost disappear. **(G2)** SS-OCT showed the corresponding vertical hyperreflective lesions almost vanished, outer retina fuzzy borders were in remission.

**Figure 3:**
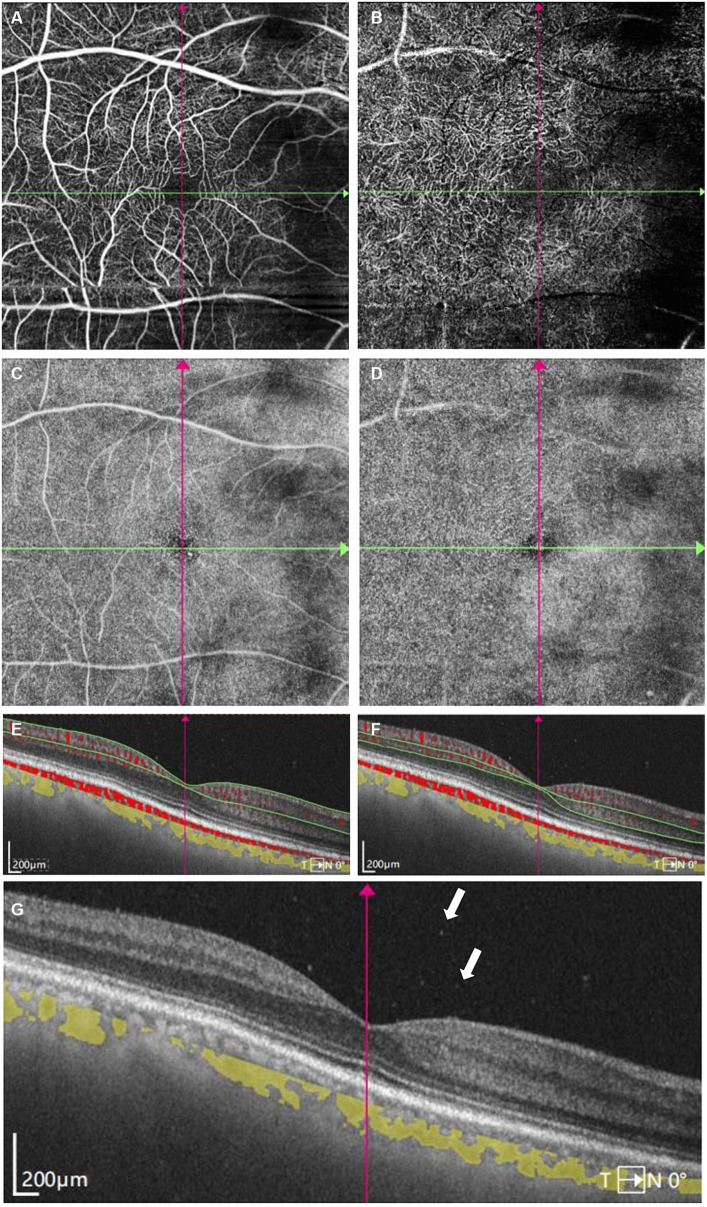
Swept-Source Optical Coherence Tomography Angiography (SS-OCTA) examinations of the right eye at initial presentation. **(A)** SS-OCTA image of superficial capillary plexus (SCP) layer. **(B)** SS-OCTA image of retinal deep capillary plexus (DCP) layer. **(C)** *Enface* SS-OCTA structural images of SCP layer. **(D)** *Enface* SS-OCTA structural images of DCP layer. **(A–D)** showed no obvious abnormality correlated with lymphoma. **(E,F)** SS-OCT B-scan centered on the fovea with flow overlay of **(E)** SCP layer and **(F)** DCP layer showed no obvious abnormal blood flow signals correlated with lymphoma. **(G)** SS-OCT showed several vitreous cells (white arrows).

A 68-year-old Chinese male presented to our hospital for worsening blurry vision in both eyes for 2 months. He reported no significant past ocular or medical history. The best-corrected visual acuity (BCVA) was 20/30 in the right and 20/60 in the left eye. The anterior segment examination revealed mild cataract of both eyes. There were no keratic precipitates or anterior chamber cells. Mild vitreous opacity with asteroid hyalosis, and several subretinal yellow lesions in the nasal peripheral fundus was observed in the right eye (Figure 1A). Moderate vitreous opacity, multiple subretinal yellow nodular lesions with varying sizes, streaky pigmentation and white exudation on the surface of several yellow subretinal nodules were detected in the left eye (Figure 1B). B-scan ultrasonography showed clusters of moderately or highly condensed punctate echoes in vitreous cavity and irregular hypoechoic lesions under the retina of the left eye (Figure 1C). SS-OCT (Ultrawide SS-OCT/OCTA, BM-400 K BMizar, TowardPi Medical Technology, Beijing, China) revealed outer retina with fuzzy borders and one vertical hyperreflective lesion in the posterior pole of fundus of the left eye (Figure 2G1). SS-OCTA image of superficial capillary plexus (SCP) layer with scanning areas of 6 mm × 6 mm showed no abnormal blood flow signals of the left eye (Figure 2A1). Multiple hyperflow signals corresponding to the vertical hyperreflective lesions on SS-OCT were unexpectedly demonstrated on the *enface* SS-OCTA images of DCP layer (Figure 2B1), which were more pronounced on the *enface* structural image of DCP layer (Figure 2D1). The *enface* structural image of SCP layer showed no abnormal reflectance correlated with lymphoma (Figure 2C1). SS-OCT B-scan with flow overlay of SCP layer and DCP layer showed inhomogenous flow signals of the vertical intraretinal hyperreflective lesion (Figures 2E1,F1). Vessel density map of SCP layer demonstrated several warm hues dots corresponding to these hyperflow spots, and they were distributed along the retinal vessels (Supplementary Figure S1). Notably, neither similar vertical hyperreflective lesions in SS-OCT nor hyperflow spots in SS-OCTA were detected in the posterior pole of the right eye (Figure 3). Homogenous hyperreflective lesions sub-RPE space in the peripheral retina could also be detected by 24 mm OCT scan (Figures 1D,E). Additionally, the aqueous humor examination of the left eye showed an elevated level of interleukin-10 (IL-10) at 21.8 pg./mL and a decreased level of IL-6 at 0.3 pg./mL, resulting in a high IL-10/IL-6 ratio of 70.4.

**Figure 4:**
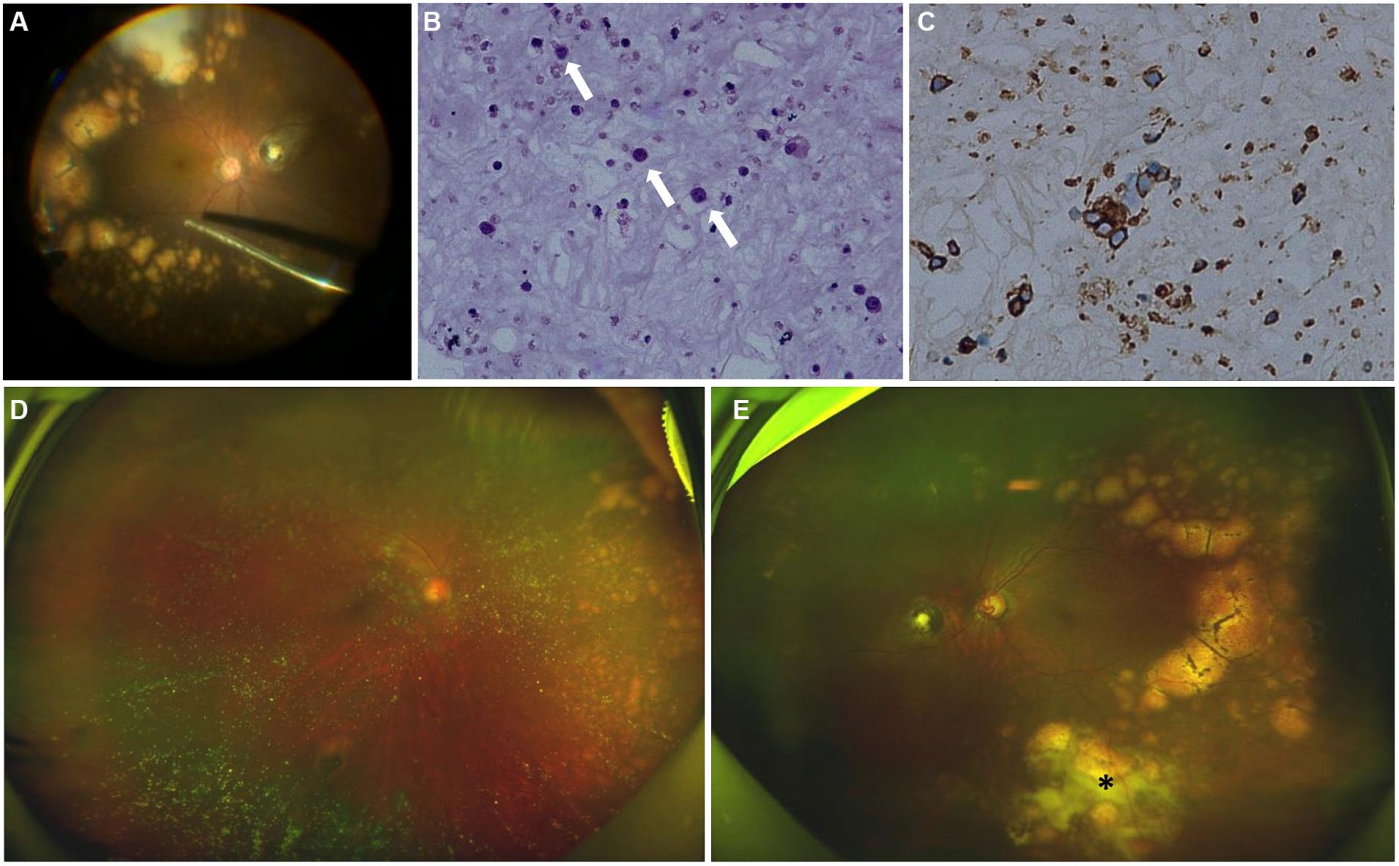
Intraoperative fundus image, cytology and immunohistochemical test of the vitreous after surgery, fundus photographs examinations after induction phase of intravitreal methotrexate injections. **(A)** Intraoperative fundus image showed multiple subretinal yellow nodular lesions with varying sizes, streaky pigmentation and white exudation (surgeo’s view). **(B)** The vitreous was infiltrated with numerous lymphocytes (white arrows) (hematoxylin and eosin ×400). **(C)** Immunohistochemical stains are positive for CD20 in the lymphocytes (peroxidase anti-peroxidase ×400). **(D)** The number and size of white-yellow subretinal lesions in the nasal retina of the right eye were reduced. **(E)** The opacity of vitreous disappeared. The number and size of white-yellow subretinal lesions were reduced obviously. White exudation (asterisk) on the surface of yellow subretinal nodules in the inferior peripheral fundus also diminished.

We strongly suspected the possibility of PVRL and performed diagnostic 23 gauge-vitrectomy for his left eye (Figure 4A). Moderate vitreous opacity was observed in the surgery, and multiple yellow nodular lesions and white exudation in the inferior retina were proved to be subretinal. Vitreous fluid was collected for cytology, immunohistochemistry and molecular analysis. Cytology unveiled atypical lymphocytes with an elevated nucleus/cytoplasm ratio and multiple prominent nucleoli (Figure 4B). Lymphoma cells in the vitreous presented positive B-cell marker of CD20, CD79a, Bcl-2 and Bcl-6, with a high Ki-67 positive rate of 30–40% (Figure 4C). There was only weak positivity of CD3, the T-cell marker. The results of IgH gene rearrangement were positive, and TCR gene rearrangement was negative in monoclonality analysis.

There were no positive findings in cranial-enhanced magnetic resonance imaging (MRI) and whole-body positron emission tomography-computed tomography (PET-CT). Thereby, a diagnosis of PVRL of both eyes was definitively confirmed.

The treatment regimen for both eyes was as follows: an induction phase of weekly intravitreal methotrexate injections for 4 weeks at a dose of 400 μg in 0.05 mL; after induction, biweekly consolidation methotrexate injections were given for 1 month at a dose of 400 μg in 0.05 mL; and a maintenance phase involves monthly methotrexate injections for 10 months. A neuro-oncological consultation was recommended to assess the necessity of prophylactic systemic chemotherapy. Following the induction phase, the patient’s BCVA improved to 20/22 of the right eye and 20/30 of the left eye. SS-OCT showed that the corresponding vertical hyperreflective lesions almost vanished. Furthermore, outer retina fuzzy borders were in remission in the left eye, and ellipsoid zone and external limiting membrane could be recognized on SS-OCT (Figures 2E2–G2). The preexisting hyperflow spots on SS-OCTA images of DCP layer almost disappeared (Figure 2B2). *Enface* SS-OCTA structural images of DCP layer also showed the preexisting hyperreflective lesions almost disappeared (Figure 2D2). SS-OCTA image of SCP layer and *enface* SS-OCTA structural images of SCP layer showed no great difference from pre-treatment images (Figures 2A2,C2). No significant changes were detected on SS-OCT in the macular area of the right eye. Fundus examination demonstrated a clear vitreous cavity postoperatively. The number and size of the peripheral white-yellow lesions were reduced obviously in both eyes, especially in the infratemporal peripheral fundus of the left eye. Moreover, the white exudation on the surface of yellow subretinal nodules in the inferior peripheral fundus of the left eye also diminished (Figures 4D,E).

The study protocol adhered to the tenets of the Declaration of Helsinki and was approved by the Medical Ethics Committee of the Second Affiliated Hospital of Zhejiang University School of Medicine, Hangzhou, China. All the clinical data were obtained from the electronic medical record system, with the patients’ consent.

## Discussion

OCT is widely used in assessing disease activity and monitoring therapeutic response in PVRL. OCT findings of PVRL include outer retina fuzzy borders, pigment epithelium detachments (PED), subretinal hyperreflective infiltration, intraretinal infiltration, subretinal fluid (SRF) and subretinal fibrosis (12). Even though pathological examination remains the gold standard for diagnosing PVRL, the specific characterizations on OCT are of great significance for the early diagnosis of PVRL. However, there are only few reported data available with SS-OCTA. SS-OCT/OCTA, distinguished by its higher resolution and broader scanning range, enhances lesion visualization in greater detail compared to traditional Spectral Domain OCT/OCTA (13). Notably, SS-OCTA can especially provide layered blood flow images with unprecedented resolution in a rapid and non-invasive way.

In our case, SS-OCT showed outer retina fuzzy borders, PED and intraretinal infiltrations, which help to establish the diagnosis of PVRL. Intraretinal infiltrations manifested as several vertical hyperreflective lesions with various width and length on SS-OCT. Most of these hyperreflective lesions extended from ganglion cell layer or inner plexiform layer to the outer layer of the neuroretina such as external limiting membrane or ellipsoid zone. Some lesions were challenging to discern due to the blurring of outer retinal boundaries. The biggest intraretinal infiltration extended from retinal nerve fiber layer to RPE, suggesting the potential for the lesion to infiltrate the entire retinal layer. Some small intraretinal infiltrations extended from inner nuclear layer to outer nuclear layer, with the majority around the outer plexiform layer. Deák et al. described the lesions as vertical hyperreflective lesions (VHRLs), which were often localized around second-order and third-order retinal vessels, varied in width but extended from the inner retina to the outermost part of the neuroretina or the RPE. Additionally, Deák et al. highlighted that VHRL could be indicative of a diagnosis of vitreoretinal lymphoma (10). In our case, some intraretinal infiltrations differed from the reported VHRLs by being shorter, thicker, and pointed like ears of wheat. Saito et al. described intraretinal infiltrations as “focal round lesions in the neural retinal layer,” some of which were larger than VHRLs and the lesions of our findings (14). Meanwhile, observations of Zhao et al. revealed that the diffuse and homogeneous hyperreflective lesions with blurred boundaries of neuroretina were also intraretinal infiltrations of PVRL (12). These diverse OCT features of intraretinal infiltrations may be able to provide clues to general infiltration patterns of malignant lymphocytes. We supposed that these microinfiltrations in our case might develop into larger lesions like VHRLs or diffuse intraretinal infiltrations along with the disease progression. Since the pathophysiology of PVRL is still not completely clear, histologic examinations of retinal tissues are imperative to elucidate the origin and infiltration mechanisms of PVRL.

No abnormal blood flow signals correlated with lymphoma were observed on the SS-OCTA image of SCP layer with scanning areas of 6 mm × 6 mm of the left eye. However, the SS-OCTA image of DCP layer showed several hyperflow spots of the left eye, which were corresponding to the vertical intraretinal microinfiltrations. The average size of these hyperflow spots were 19,606 ± 14,412 um^2^ (range: 2100–60,000 um^2^) on the SS-OCTA image of DCP layer. All the hyperflow spots located in the perifoveal area. Remarkably, the study revealed that a majority of the intraretinal microinfiltrations (15, 65.2%) were situated directly beneath superficial retinal vessels within the retinal nerve fiber layer. Conversely, there were no discernible vessels overlaying the remaining intraretinal microinfiltrations (8, 34.8%). However, there were apparent smaller blood flow signals originating from the outer plexiform layer around them (Supplementary Figure S2). Moreover, the size of intraretinal microinfiltrations was unrelated to their positioning beneath retinal vessels.

SS-OCT with flow overlay also demonstrated inhomogenous flow signals of the vertical intraretinal hyperreflective lesions. It was challenging to distinguish the boundary of flow signals overlaying the vertical intraretinal hyperreflective lesions from the flow signals of retinal vessels above them. Among these lesions, 2 (8.7%) exhibited blood flow signals above the lesion, 3 (13.0%) presented scattered blood flow signals, 6 (26.1%) showed blood flow signals centrally, and 11 (47.8%) displayed blood flow signals laterally. Additionally, one lesion (4.3%) exhibited no significant blood flow distribution.

It is well documented that the migration of lymphocytes within the nervous tissues relies on a selective interaction between adhesion molecules on lymphocytes and the vascular endothelium of the central nervous system (15). Chen et al. described hyperreflective lesions along the vessels on the mid-retinal slabs of *enface* OCTA as perivascular flower-bud-like lesions (PFBLs) in 34.3% of PVRL cases. They also found that vessels with PFBLs showed higher reflectance than the surrounding retina, with the clear hyporeflective vascular wall boundary absent. They posited that PFBLs could potentially represent lymphoma deposits originating from retinal vascular microinfiltrations, given their proximity to retinal vessels (11). Intraretina hyperreflective infiltrations with hyperflow signals reported in our case manifested a similar hyperreflective appearance to PFBLs on *enface* SS-OCTA structural images of DCP layers. Additionally, we also noticed that the hyporeflective vascular wall boundary of vessels above intraretinal infiltrations was absent on SS-OCT before treatment (Figure 2G1), implying the infiltration of vascular wall. However, there were still some notable differences between the two lesions. Primarily, all PFBLs were situated along the vessels on *enface* SS-OCTA. While we observed that certain intraretinal microinfiltrations were not located along retinal vessels in the retinal nerve fiber layer. In addition, no hyperflow signals were detected in PFBLs by SS-OCTA, while hyperflow signals were obvious in the intraretinal microinfiltration lesions on DCP layer in our case. Moreover, these intraretinal infiltrations located close to retinal vessels from retinal nerve fiber layer to the outer plexiform layer, thus we suppose that these intraretinal microinfiltrations could potentially result from hematological spread from surrounding infiltrated vessels. The microenvironment with sufficient blood supply from retinal vessels may play a pivotal role in the growth, invasion and metastasis of malignant lymphocytes. That may also explain why intraretinal microinfiltrations mostly located around retinal vessels. Pierro et al. reported a case of a patient affected by PVRL with multiple subretinal hyperreflective infiltrates on OCT, and reported that several hyperreflective lesions were also detected on OCTA structural images (16). To our knowledge, few articles have previously observed hyperflow signals on SS-OCTA images of DCP layers in patients with PVRL. The current pathological results are still not sufficient to elucidate the relationship between PVRL and retinal vascular infiltration. Passarin et al. reported a case of brain intravascular lymphomatosis presenting hemorrhage of brain vessels. They postulated that the mechanism of hemorrhage might be chronic degenerative or inflammatory changes of the vessel wall with lymphomatosis (17). we excluded images with artifacts that might affect the evaluations in our case. Moreover, we calculated the diameter of the retinal vessels above the microinfiltration lesions on SCP layer. The average diameter of these retinal vessels decreased from 54.27 ± 13.08 um (range: 33–72 um) to 48.73 ± 8.51 um (range: 28–59 um) after methotrexate intravitreal injections. Hence, we hypothesize that the interaction between lymphocytes and vascular endothelial cells could potentially induce chronic damage to the vascular wall, resulting in the dilation of retinal vessels. The abnormal hyperflow signals could stem from the dilation of infiltrated retinal vessels. Histologic examinations of retinal tissues may be essential in elucidating the pathological mechanism associated with the hyperflow signals.

In the left eye, multiple hyperreflective lesions on *enface* SS-OCTA structural images of DCP layers were also detected to be co-localized with the vertical hyperreflective lesions (Figure 2D1). These lesions presented as punctations of varying sizes. Larger lesions displayed as irregular masses with spiculated sign, which were morphologically different from the roundish PFBLs. The spiculated sign on SS-OCTA seemed to demonstrate the metastasis characteristic of infiltrating tumor cells into surrounding normal tissues more vividly. The average size of hyperreflective lesions on *enface* SS-OCTA structural images was 55,561 ± 86,184 um^2^ (range: 5900–93,000 um^2^), which was significantly larger than those observed on SS-OCTA images of DCP layer. This could be attributed to the fact that *enface* SS-OCTA structural images displayed the actual size of intraretinal microinfiltrations, whereas SS-OCTA images only depicted the blood flow signals within these lesions. Moreover, these lesions were reduced and the spiculated sign was in remission after intravitreal methotrexate injections. This suggests that observation of intraretinal microinfiltration lesions on SS-OCTA might also play a crucial role in monitoring the therapeutic efficacy of PVRL. Since there was no interference of retinal vessel signals, microinfiltration lesions seemed to be much more intuitional on *enface* SS-OCTA structural images than that on angiographic images (Figures 2B1,D1).

Interestingly, none of the intraretinal microinfiltration lesions were detectable on the wide-angle fundus photography, infrared or fluorescein angiography fundus images. We supposed that these lesions might be an ultra-early prediction of PVRL infiltration, implying that the intraretinal microinfiltration lesions might evolve into visible lesions on fundus along with the disease progression. SS-OCTA was sensitive and noninvasive to detect the occurrence and development of the intraretinal microinfiltration lesions, thus we believe that it would be a feasible approach for early diagnosis and monitoring the therapeutic efficacy of PVRL. Intraretinal hyperflow microinfiltration lesions exhibited by SS-OCTA could also act as significant biomarkers to evaluate the activity of PVRL. Further investigations are needed to reveal the natural development process of intraretinal infiltrations.

There were several limitations. First, the vessel diameter was manually measured using the built-in measurement tool in OCTA device. Due to the indistinct vessel boundary, measurement error should be considered when vessel diameter was compared before and after treatment. Histological examination is essential for elucidating the morphology of infiltrated retinal vessels. Second, hyperreflective intraretinal lesion was previously detected in SS-OCT in uveitis cases such as sarcoidosis (18), whose OCTA manifestation has seldom been reported. It is uncertain whether the hyperflow signals in the microinfiltration lesion was typical character of PVRL. Hence, More PVRL cases were required to verify the dilation of infiltrated retinal vessels over the microinfiltration lesion, and further clarify the value of hyperflow signals in differential diagnosis and activity assessment.

## Conclusion

SS-OCTA has been increasingly used in assessing the fundus disease due to its superiority in resolution, depth and width. This report characterized intraretinal microinfiltrations of PVRL using wide-field SS-OCTA. Those distinctive lesions presented as vertical hyperreflective lesions in SS-OCT, locating close to retinal vessels from the retinal nerve fiber layer to the outer plexiform layer, exhibiting a concentrated distribution in the macular region. Notably, traditional fundus photography or fluorescein angiography failed to detect these lesions. Intriguingly, SS-OCTA images of DCP layer demonstrated a number of punctate hyperflow lesions corresponding to those intraretinal microinfiltrations lesions, which were supposed to be the dilation of the infiltrated retinal vessels. The microinfiltrations exhibited sensitivity to intraocular chemotherapy of methotrexate, rapidly disappearing after the fourth injection. The Intraretinal hyperflow microinfiltration lesions detected by SS-OCTA would act as significant biomarkers to evaluate activity of PVRL. Furthermore, SS-OCTA can clearly demonstrate the location, size and variation of microinfiltration lesions. This underscores the potential of SS-OCTA in the early diagnosis of PVRL microinfiltrations and the monitoring of therapeutic responses.

## Data availability statement

The original contributions presented in the study are included in the article/Supplementary material, further inquiries can be directed to the corresponding author.

## Ethics statement

The studies involving humans were approved by Medical Ethics Committee of the Second Affiliated Hospital of Zhejiang University School of Medicine, Hangzhou, China. The studies were conducted in accordance with the local legislation and institutional requirements. The participants provided their written informed consent to participate in this study. Written informed consent was obtained from the individual(s) for the publication of any potentially identifiable images or data included in this article.

## Author contributions

ZX: Conceptualization, Data curation, Formal analysis, Investigation, Methodology, Writing – original draft, Writing – review & editing. HB: Data curation, Formal analysis, Investigation, Methodology, Writing – original draft, Writing – review & editing. XL: Writing – review & editing, Formal analysis. JS: Data curation, Investigation, Writing – original draft. YS: Investigation, Writing – original draft, Formal analysis. YW: Funding acquisition, Project administration, Resources, Writing – review & editing, Conceptualization, Investigation, Methodology.

## Supplementary material

SUPPLEMENTARY Figure S1 Vessel density map of superficial capillary plexus layer of the left eye before induction phase of intravitreal methotrexate injections. Several warm hues dots were distributed along the retinal vessels.

SUPPLEMENTARY Figure S2 Another typical intraretinal hyperflow microinfiltration lesions on Swept-Source Optical Coherence Tomography Angiography (SS-OCTA) with scanning areas of 6mm × 6mm of the left eye **(A1–G1)** before and **(A2–G2)** after induction phase of intravitreal methotrexate injections. **(A1)** SS-OCTA image of superficial capillary plexus (SCP) layer showed no abnormal blood flow signals correlated with lymphoma. **(B1)** SS-OCTA image of retinal deep capillary plexus (DCP) layer showed several hyperflow spots. **(C1)** *Enface* SS-OCTA structural images of SCP layer showed no abnormal reflectance correlated with lymphoma. **(D1)** *Enface* SS-OCTA structural images of DCP layer showed multiple hyperreflective lesions. **(E1,F1)** SS-OCT B-scan with flow overlay of **(E1)** SCP layer and **(F1)** DCP layer showed flow signals of the small vertical intraretinal hyperreflective lesion. There were apparent smaller blood flow signals originating from the outer plexiform layer around it. **(G1)** SS-OCT showed outer retina with fuzzy borders and the vertical hyperreflective lesion extended from inner nuclear layer to outer nuclear layer. **(A2)** SS-OCTA image of SCP layer showed no great difference from A1. **(B2)** SS-OCTA image of DCP layer showed the preexisting hyperflow spots almost disappeared. **(C2)** *Enface* SS-OCTA structural images of SCP layer showed no great difference from **C1**. **(D2)** *Enface* SS-OCTA structural images of DCP layer showed the preexisting hyperreflective lesions almost disappeared. **(E2,F2)** SS-OCT B-scan with flow overlay of **(E2)** SCP layer and **(F2)** DCP layer showed the preexisting hyperflow signals of the vertical intraretinal hyperreflective lesion almost disappear. **(G2)** SS-OCT showed the corresponding vertical hyperreflective lesions almost vanished, outer retina fuzzy borders were in remission.
