# Supplementary figures and images for: Case report: Intraretinal hyperflow microinfiltration lesions on swept-source optical coherence tomography angiography as a potential biomarker of primary vitreoretinal lymphoma
Source: Front Med (Lausanne). 2024 Apr 26;11:1386979. doi: 10.3389/fmed.2024.1386979 (PMC11082336; doi:10.3389/fmed.2024.1386979)

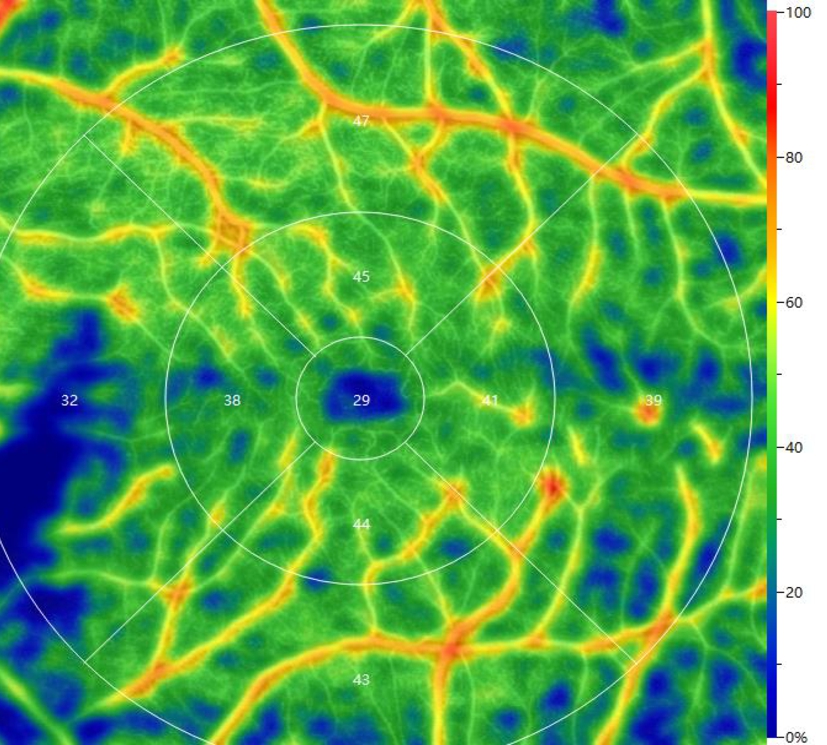

Supplement: SUPPLEMENTARY Figure S1 — Vessel density map of superficial capillary plexus layer of the left eye before induction phase of intravitreal methotrexate injections. Several warm hues dots were distributed along the retinal vessels. [file Image_1.JPEG]

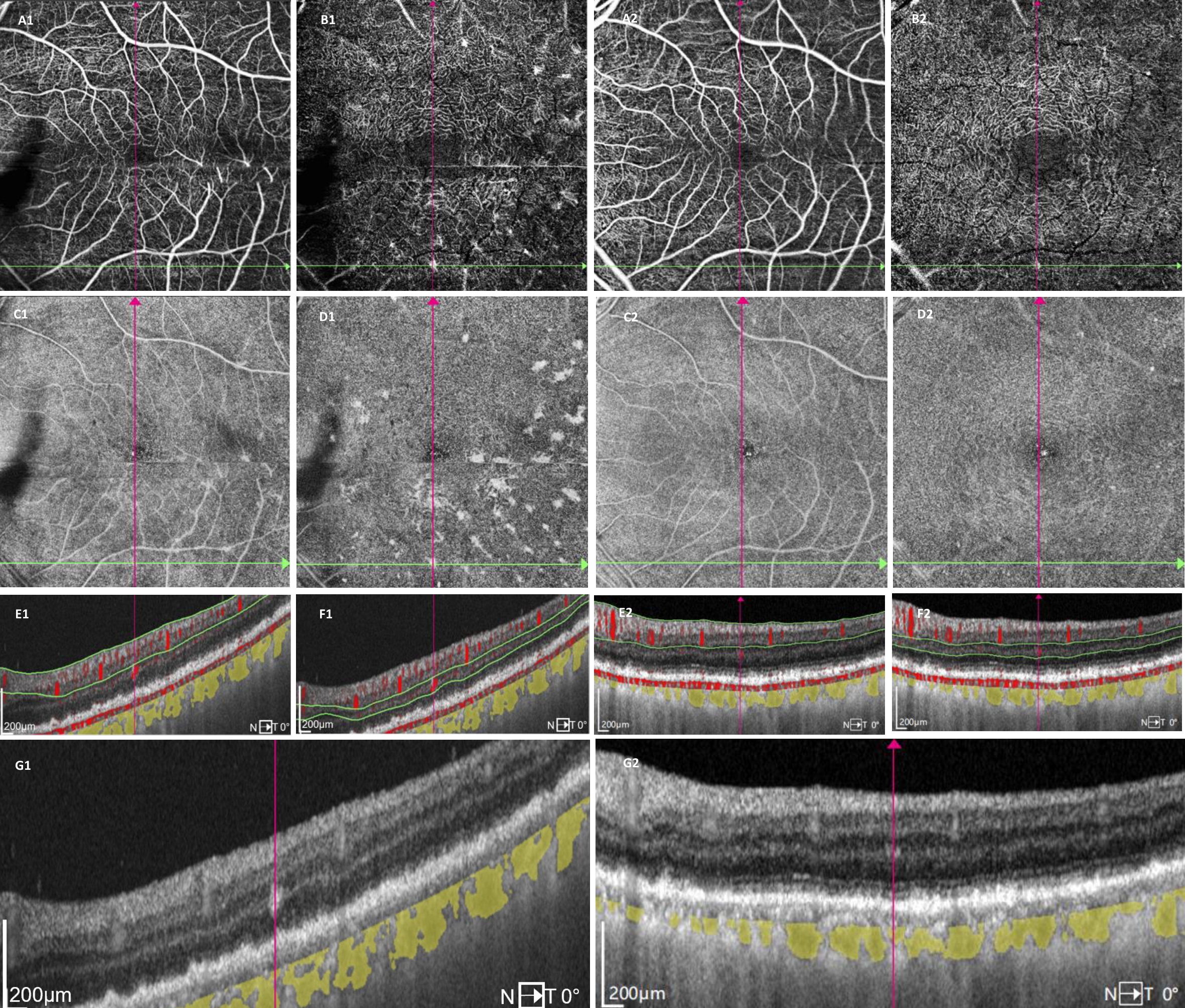

Supplement: SUPPLEMENTARY Figure S2 — Another typical intraretinal hyperflow microinfiltration lesions on Swept-Source Optical Coherence Tomography Angiography (SS-OCTA) with scanning areas of 6mm × 6mm of the left eye (A1–G1) before and (A2–G2) after induction phase of intravitreal methotrexate injections. (A1) SS-OCTA image of superficial capillary plexus (SCP) layer showed no abnormal blood flow signals correlated with lymphoma. (B1) SS-OCTA image of retinal deep capillary plexus (DCP) layer showed several hyperflow spots. (C1) Enface SS-OCTA structural images of SCP layer showed no abnormal reflectance correlated with lymphoma. (D1) Enface SS-OCTA structural images of DCP layer showed multiple hyperreflective lesions. (E1,F1) SS-OCT B-scan with flow overlay of (E1) SCP layer and (F1) DCP layer showed flow signals of the small vertical intraretinal hyperreflective lesion. There were apparent smaller blood flow signals originating from the outer plexiform layer around it. (G1) SS-OCT showed outer retina with fuzzy borders and the vertical hyperreflective lesion extended from inner nuclear layer to outer nuclear layer. (A2) SS-OCTA image of SCP layer showed no great difference from A1. (B2) SS-OCTA image of DCP layer showed the preexisting hyperflow spots almost disappeared. (C2) Enface SS-OCTA structural images of SCP layer showed no great difference from C1. (D2) Enface SS-OCTA structural images of DCP layer showed the preexisting hyperreflective lesions almost disappeared. (E2,F2) SS-OCT B-scan with flow overlay of (E2) SCP layer and (F2) DCP layer showed the preexisting hyperflow signals of the vertical intraretinal hyperreflective lesion almost disappear. (G2) SS-OCT showed the corresponding vertical hyperreflective lesions almost vanished, outer retina fuzzy borders were in remission. [file Image_2.JPEG]
